# *AtGATA5* contributes to ABA-mediated seed germination by promoting *NCED3* and *ABI4* expression in *Arabidopsis thaliana*

**DOI:** 10.1080/15592324.2026.2687956

**Published:** 2026-06-11

**Authors:** Byeonggyu Kim, Kihwan Kim, Won-Chan Kim

**Affiliations:** a Department of Applied Biosciences, Kyungpook National University, Daegu, Republic of Korea; b Upland Field Machinery Research Center, Kyungpook National University, Daegu, Republic of Korea; c Department of Integrative Biology, Kyungpook National University, Daegu, Republic of Korea; d NGS Core Facility, Kyungpook National University, Daegu, Republic of Korea

**Keywords:** *Arabidopsis thaliana*, *AtGATA5*, abscisic acid, seed germination, *NCED3*, *ABI4*

## Abstract

Abscisic acid (ABA) is a key phytohormone that regulates seed germination, stomatal closure, and responses to abiotic stresses. The biosynthesis and signaling of ABA are controlled by a complex gene network, with *NCED3* serving as the rate-limiting enzyme in ABA biosynthesis and *ABI4* acting as a central transcription factor in ABA signaling. However, upstream regulators that coordinately control both processes during seed germination remain incompletely understood. In this study, we investigated the role of *AtGATA5*, a GATA transcription factor in *Arabidopsis thaliana*, in ABA-mediated seed germination. To assess the germination phenotype under ABA treatment and the expression of ABA-related genes, we performed a transient activity assay (TAA) using *Arabidopsis* protoplasts and gene expression analysis in transgenic plants. *AtGATA5*-overexpression (*AtGATA5*-OX) plants exhibited significantly reduced germination rates under exogenous ABA compared with wild type (WT), and *gata5* mutant, generated by CRISPR/Cas9-mediated genome editing, showed a similar ABA-hypersensitive phenotype. The expression levels of *NCED3* and *ABI4* were markedly elevated in *AtGATA5*-OX plants, and TAA confirmed that AtGATA5 upregulates the promoter activities of both *NCED3* and *ABI4*. These findings suggest that *AtGATA5* plays a functional role in ABA-mediated seed germination by modulating *NCED3* and *ABI4* expression, and that AtGATA5 may represent a novel regulator of ABA homeostasis in plants.

## Introduction

Abscisic acid (ABA) is a sesquiterpenoid phytohormone that plays a central role in plant growth and development, and stress response.^1^ ^,^ ^2^ ABA is a key regulator of seed dormancy and germination, stomatal movement, and adaptive responses to abiotic stresses including drought, salinity, and cold.^2-4^ During seed germination, ABA acts as a negative regulator inhibiting germination and promoting dormancy, while gibberellin (GA) antagonizes ABA to promote germination.^5^ ^,^ ^6^ The balance between ABA and GA levels is a key determinant of germination timing and efficiency.^7^ ^,^ ^8^ Given its broad regulatory functions, understanding the transcriptional mechanisms that control ABA biosynthesis and signaling has significant implications for improving crops stress tolerance and seed quality.^4^ ^,^ ^9^

ABA biosynthesis proceeds through the oxidative cleavage of C40 epoxycarotenoids by the 9-cis-epoxycarotenoid dioxygenase (NCED) family of enzymes.^5^ ^,^ ^10^ Among these, *NCED6* and *NCED9* are the primary isoforms responsible for ABA accumulation in seeds during dormancy,^11^ ^,^ ^12^ while *NCED3* is predominantly associated with ABA biosynthesis in response to drought and osmotic stress in *Arabidopsis.* ^13^ ^,^ ^14^ However, *NCED3* expression has also been shown to be upregulated by ABI4 during seed germination through a positive regulatory feedback loop that further amplifies ABA biosynthesis and reinforces dormancy.^15^ Upregulation of *NCED3* can therefore elevate endogenous ABA content in germinating seeds and contribute to germination inhibition, making *NCED3* a functionally relevant component of ABA accumulation during seed germination regardless of its primary association with drought responses. In the ABA signaling pathway, ABA INSENSITIVE 4 (ABI4), an AP2/ERF domain transcription factor, serves as a key mediator of ABA responses during seed germination and early seedling development.^16^ ^,^ ^17^ ABI4 represses germination by suppressing GA-responsive genes while activating ABA-responsive elements.^17-19^ ABI4 also functions in coordination with other ABI-class regulators such as ABI5 to suppress post-germination growth.^20^ Upstream of ABI4, the core ABA signaling module comprising PYR/PYL receptors, PP2C phosphatases, and SnRK2 kinases has been well characterized.^9^ ^,^ ^21-23^ Nevertheless, the transcription factors that coordinately regulate both *NCED3* and *ABI4* to modulate ABA levels during seed germination remain poorly characterized.^2^ ^,^ ^6^ ^,^ ^24^

GATA transcription factors carry a conserved zinc-finger domain that recognizes the (A/T)GATA(A/G) sequence motif and found in plants, animals, and fungi.^25-27^ In *Arabidopsis*, the GATA family comprise 29 members.^25^ ^,^ ^28^ Their expression levels are controlled by the circadian clock and influenced by light, leading to developmental changes such as alterations in flowering time, hypocotyl elongation, and phytohormone biosynthesis.^29-31^ GATA transcription factors regulate diverse processes including chloroplast development and nitrogen assimilation.^28^ ^,^ ^29^ Among the functionally characterized members, *GATA25* regulates flowering time^30^ and hypocotyl elongation^31^ through gain- and loss-of-function studies, and the poplar ortholog PtrGATA9 facilitates interfascicular fiber cell differentiation through activation of NAC-domain transcription factors.^32^ Despite this growing body of evidence, the roles of GATA transcription factors in ABA-mediated stress responses and seed germination remain largely uncharacterized.

*AtGATA5* is a member of the *Arabidopsis* GATA transcription factor family. *AtGATA5* is known to be a light-responsive,^33-35^ and was recently reported to positively regulate NAC-domain transcription factors, the master regulators of secondary cell wall (SCW) biosynthesis, thereby influencing SCW formation and leaf morphology.^36^ ^,^ ^37^ Notably, *Arabidopsis thaliana* seeds are positively photoblastic and require light as a permissive signal for germination. Light signaling promotes GA biosynthesis and reduces ABA content, thereby facilitating germination.^5^ ^,^ ^7^ Given that GATA transcription factors, including *AtGATA5*, are regulated by the circadian clock and influenced by light,^33-35^ and that ABA biosynthesis is also subject to light and circadian regulation, it is conceivable that *AtGATA5* may connect light and circadian signals to the ABA-mediated control of seed germination. We therefore hypothesize that *AtGATA5* plays a role in promoting ABA accumulation during seed germination by upregulating the expression of *NCED3* and *ABI4*, and that this regulatory function may be related to light and circadian regulation. However, the precise role of *AtGATA5* in ABA-mediated regulation of seed germination has not been investigated.

In this study, we demonstrate that *AtGATA5* plays an important role in ABA-mediated seed germination by modulating the expression of *NCED3* and *ABI4*. We generated *AtGATA5*-OX transgenic plants and CRISPR/Cas9-mediated knock-out mutants (*gata5*) to investigate germination phenotypes under ABA treatment. TAA using *Arabidopsis* protoplasts and gene expression analysis in transgenic plants were conducted to characterize the regulatory relationship between *AtGATA5* and target genes.

## Material and methods

### Plant materials and growth conditions

*A. thaliana* ecotype Columbia (Col-0) was used in this study. Seeds were surface-sterilized for 10 min in 0.15% NaClO and 5 s in 70% ethanol, rinsed eight times with sterile water, and cold-treated for 3 d at 4 °C in the dark.^1^ Seeds were germinated on 1/2 MS medium including multiple vitamins, 6 mM MES, and 1% sucrose. Plants were grown under long-day (LD) conditions (16 h light/8 h dark photoperiod) at 23 °C.

### RNA isolation and gene expression analysis

Total RNA was extracted using the TRIzol reagent (Invitrogen) and RNA purity was confirmed by NanoDrop spectrophotometry prior to downstream analyses. Genomic DNA contamination was eliminated by treatment with RNases-Free DNase (QIAGEN), and complete removal was verified by PCR using *ACTIN8* (At1g49240)-specific primers on DNase-treated RNA in the absence of reverse transcription. Two micrograms of DNase-treated total RNA were reverse-transcribed using Superscript III reverse transcriptase in 20 μL reactions, and successful cDNA synthesis was confirmed by PCR amplification of *ACTIN8* followed by agarose gel electrophoresis. For real-time PCR analysis, gene expression levels were quantified using SYBR green to monitor double-stranded DNA synthesis. Primer specificity was confirmed by melting curve analysis, with all primer pairs yielding a single dissociation peak, and the qPCR system was calibrated prior to use. Cycle threshold (*C_T_*) values for target genes were normalized to the *C_T_* value of *ACTIN8* to enable comparison across samples.^38^ All experiments were conducted with three independent biological replicates.

### Transient activity assay

Protoplast isolation and PEG-mediated transfection were performed as previously described.^39^ Protoplasts were isolated from 3-week-old WT or *gata5* plants. An effector plasmid expressing *AtGATA5* under the CaMV 2 × 35S promoter was co-transfected with a reporter plasmid the promoter region of *NCED3* (−1650 to −1 bp) or *ABI4* (−1575 to −1 bp) fused to the β*-*glucuronidase (GUS) reporter gene. Protoplasts transfected with only the reporter plasmid served as controls. After 12 h incubation, protoplasts were lysed and analyzed for GUS activity. All experiments were conducted with three independent biological replicates.

### Generation of the *gata5* mutant by CRISPR/Cas9 system

A single-guide RNA (sgRNA) targeting the exon of *AtGATA5* was designed and cloned into a binary vector for CRISPR/Cas9-mediated genome editing.^40^ ^,^ ^41^ The construct was introduced into *A. thaliana* by *Agrobacterium tumefaciens*-mediated transformation using the floral dip method.^42^ Transgenic T1 plants were selected on 1/2 MS medium containing kanamycin. T2 generation plants were screened by the T7 Endonuclease Ⅰ (T7E1) assay to identify homozygous mutant lines, and Sanger sequencing was subsequently performed on the selected homozygous plants to confirm the precise sequence alteration. T3 generation plants were used for all subsequent analyses. A single nucleotide insertion within *AtGATA5* coding sequence was identified, causing a frameshift and premature stop codon, predicting a truncated non-functional protein.

### Seed germination ratio analysis

Surface-sterilized seeds of WT, *AtGATA5*-OX, and *gata5* were plated on 1/2 MS medium supplemented with 0, 0.25, 0.5, or 1 μM ABA.^1^ ^,^ ^7^ After stratification at 4 °C for 3 d, plates were transferred to a growth chamber. Germination was scored daily for 4 d, with seeds considered germinated upon radicle emergence through the seed coat. Germination rates were calculated as the percentage of germinated seeds out of the total seeds plated. At least three independent biological replicates were performed, with 200 seeds per replicate.

### Statistics

Data are presented as mean ± standard deviation (SD) of at least three independent biological replications. Difference was considered to be statistically significant when the *p*-value was less than 0.1 using a pairwise Student's *t*-test.

## Results

### CRISPR/Cas9-mediated generation and confirmation of the *gata5* mutant

To investigate the *in vivo* function of *AtGATA5* in the ABA pathway, we generated a loss-of-function mutant of *AtGATA5* using the CRISPR/Cas9 genome-editing system. A single-guide RNA (sgRNA) targeting the exon of *AtGATA5* was introduced into WT *A. thaliana* by the floral dip method. Sequence analysis of T3 generation homozygous plants revealed a single nucleotide insertion within the coding sequence of *AtGATA5*, causing a frameshift and premature stop codon (Figure S1A). As a result, the WT amino acid sequence (FTEYSGPNLTGTPTE) was converted to an aberrant truncated sequence (FTEYSGPNLHRNPD*), predicting a non-functional AtGATA5 protein. Homozygosity of the mutation was confirmed by the T7 Endonuclease Ⅰ (T7EⅠ) assay (Figure S1B), and line #5 was selected for all subsequent analysis.

### *AtGATA5*-OX and *gata5* mutant exhibit enhanced sensitivity to ABA during seed germination

**Figure 1.**
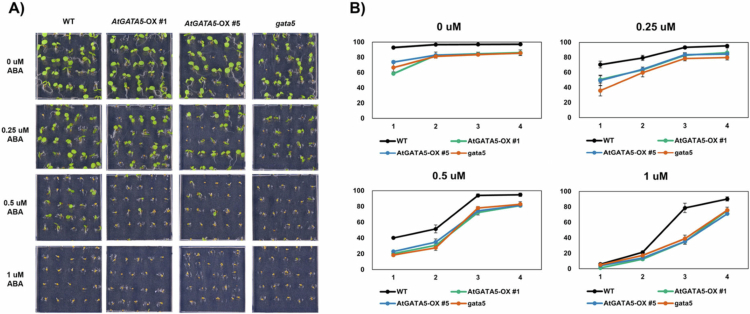
ABA hypersensitivity of *AtGATA5*-OX and *gata5* mutants during seed germination. (a) Seeds from WT, *AtGATA5*-OX #1, *AtGATA5*-OX #5, and *gata5* mutants were germinated on 1/2MS medium supplemented with 0, 0.25, 0.5, or 1 μM ABA and scored 4 d after stratification. (b) Germination fates of WT, *AtGATA5*-OX #1, *AtGATA5*-OX #5, and *gata5* seeds were recorded daily for 4 d on medium containing 0, 0.25, 0.5, or 1 μM ABA. Data are presented as mean ± SD (*n* = 3 replicates, 200 seeds per replicate).

To investigate the role of AtGATA5 in ABA-mediated seed germination, we compared the germination phenotypes of WT, *AtGATA5*-overexpressing lines (*AtGATA5*-OX #1 and #5), and the *gata5* loss-of-function mutant on 1/2MS plate supplemented with various concentrations of ABA (Figure 1A). On plate without ABA, all genotypes reached high germination rates by the end of the observation period, although *AtGATA5*-OX and *gata5* showed slightly delayed germination compared with WT, which may reflect an elevated basal ABA content or altered ABA sensitivity in these lines, consistent with their elevated NCED3 expression. Under exogenous ABA treatment, the germination rates of *AtGATA5*-OX and *gata5* were markedly lower than those of WT at all concentrations tested, and this difference became progressively more pronounced as the ABA concentration increased (Figure 1B), indicating that the additional reduction in germination is attributable to exogenous ABA treatment. The observation that both overexpression and loss-of-function of *AtGATA5* confer ABA hypersensitivity suggests that the precise expression level of AtGATA5 is critical for maintaining proper ABA responsiveness during seed germination.

### *NCED3* and *ABI4* are upregulated in *AtGATA5*-OX plants

**Figure 2.**
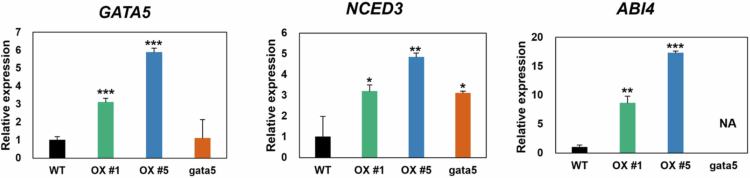
Expression analysis of *AtGATA5*, *NCED3*, and *ABI4* were analyzed by qRT-PCR in WT, *AtGATA5*-OX #1, *AtGATA5*-OX #5, and *gata5* mutants. Expression levels were normalized to *ACTIN8* and presented relative to WT. *ABI4* expression level was not detected in *gata5* mutants. Data are presented as mean ± SD (*n* = 3 replicates). Asterisks indicate statistically significant differences from control by using Student's *t*-test (***p < 0.001, **p < 0.05, *p < 0.1).

To examine whether the expression of ABA-related genes was altered in response to changes in *AtGATA5* levels, we analyzed the expression of *NCED3* and *ABI4* by quantitative reverse transcription PCR (qRT-PCR) in WT, *AtGATA5*-OX, and *gata5* mutants. Consistent with the transgenic design, *AtGATA5* gene expression levels were significantly elevated in both *AtGATA5*-OX plants compared with WT. In the *gata5* mutant, *AtGATA5* mRNA levels were comparable to those in WT (Figure 2), which is consistent with the nature of the CRISPR/Cas9-induced mutation, in which a frameshift and premature stop codon disrupt protein function without affecting transcription at the *AtGATA5* locus. Notably, *NCED3* expression was also elevated in the *gata5* mutant, although the mechanism underlying this elevation remains to be determined (Figure 2). The expression of *ABI4*, a central transcription factor in ABA signaling, was markedly up-regulated in *AtGATA5*-OX compared with WT, whereas *ABI4* expression was not detected in the *gata5* mutant (Figure 2). These results suggest that *AtGATA5* contribute to the transcriptional activation of *ABI4*, and that increased expression of *AtGATA5* is associated with elevated levels of both *NCED3* and *ABI4* in plants.

### AtGATA5 positively regulates *NCED3* and *ABI4* activities

**Figure 3.**
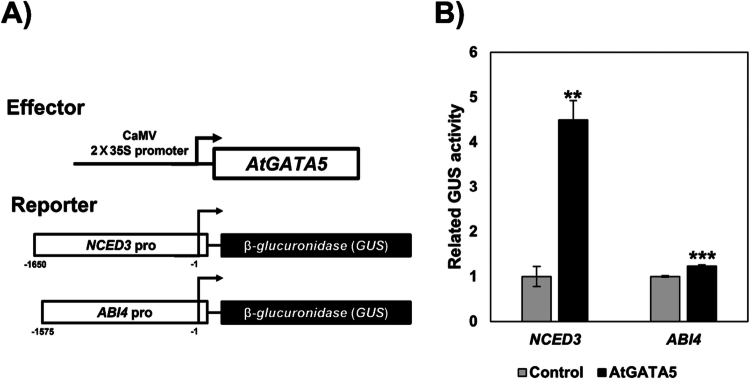
Transient activity assay *NCED3* and *ABI4*. (a) Schematic diagram of effector and reporter construct used this transient activity assay. (b) Transient activity assay showing *AtGATA5* upregulate *NCED3* and *ABI4*. Protoplasts transfected only reporter construct was using as control. Data are presented as mean ± SD (*n* = 3 replicates). Asterisks indicate statistically significant differences from control by using Student's *t*-test (***p < 0.001, **p < 0.05, *p < 0.1).

To examine whether AtGATA5 can modulate the promoter activities of *NCED3* and *ABI4*, a TAA was performed as described in the Materials and methods (Figure 3A). The results demonstrated that the promoter activities of both *NCED3* and *ABI4* were significantly increased in the presence of the AtGATA5 effector compared with the control (Figure 3B). These results indicate that AtGATA5 can upregulate the promoter activities of both *NCED3* and *ABI4*, and support a role for AtGATA5 in the regulation of ABA-related gene expression.

### *AtGATA5*-dependent activation of *NCED3* and *ABI4* is confirmed in the *gata5* background

**Figure 4.**
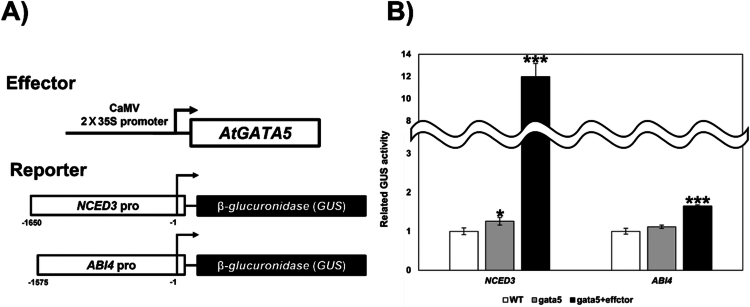
AtGATA5 restores *NCED3* and *ABI4* activities in *gata5* protoplasts. (a) Schematic diagram of effector and reporter construct used this transient activity assay. (b) Relative GUS activity driven by the *NCED3* or *ABI4* in protoplasts from WT and *gata5*, with or without co-transfected of the AtGATA5 effector construct. Protoplasts transfected with only the reporter construct were used as control. Data are presented as mean ± SD (*n* = 3 replicates). Asterisks indicate statistically significant differences from control by using Student's *t*-test (***p < 0.001, **p < 0.05, *p < 0.1).

To further examine *AtGATA5*-mediated regulation of *NCED3* and *ABI4* promoter activities, a TAA was conducted using protoplasts isolated from WT and *gata5* mutant as described in the Materials and Methods. In *gata5* protoplasts, *NCED3* promoter activity was slightly elevated compared with WT, while *ABI4* promoter was similar WT. In *gata5* protoplasts transfected with *AtGATA5* effector, the promoter activities of both *NCED3* and *ABI4* were recovered (Figure 4B). These results indicated that the promoter activities of *NCED3* and *ABI4* are responsive to AtGATA5 and support a role for *AtGATA5* in the regulation of these genes.

## Discussion

We identified *AtGATA5* as a novel regulator of ABA-mediated seed germination in *A. thaliana*. Our results demonstrate that *AtGATA5* is associated with elevated expression of *NCED3* and *ABI4*, key components of ABA biosynthesis and signaling, respectively, and that the presence of AtGATA5 promotes their promoter activities as assessed by transient activity assay (TAA). These findings suggest that *AtGATA5* functions upstream of both ABA biosynthesis and signal transduction during seed germination, although the precise molecular mechanism underlying this regulation remains to be determined.

A notable feature of this study is that both *AtGATA5*-OX and *gata5* plants exhibited ABA hypersensitivity during seed germination, with reduced germination rates compared with WT across all ABA concentrations tested (Figure 1). This bidirectional phenotype, in which both overexpression and loss-of-function of a single transcription factor confer a similar hypersensitive phenotype, suggests that the precise dosage of *AtGATA5* is critical for maintaining appropriate ABA responsiveness.^2^ ^,^ ^6^ ^,^ ^21^ ^,^ ^22^ We propose that *AtGATA5* operates within a finely balanced transcriptional network governing ABA homeostasis, whereby deviations in either direction from the optimal expression level perturb the regulatory equilibrium and sensitize seeds to ABA. This is consistent with the tight quantitative control characterizing the PYR/PYL–PP2C–SnRK2 ABA signaling module.^9^ ^,^ ^23^

The elevated expression of *NCED3* in *AtGATA5*-OX plants (Figure 2) is consistent with the ABA hypersensitivity phenotype, as NCED3 is the rate-limiting enzyme in ABA biosynthesis and its overexpression increases endogenous ABA levels.^10^ ^,^ ^13^ ^,^ ^14^ Upregulation of *NCED3* in *AtGATA5*-OX could therefore elevate basal ABA content, further sensitizing germinating seeds to exogenous ABA.^43^ This interpretation is consistent with our TAA data showing that AtGATA5 enhances *NCED3* promoter activity (Figure 3), suggesting that the regulation occurs at the transcriptional level. Transcriptional control of ABA biosynthesis genes has been proposed as a key mechanism for maintaining ABA homeostasis in seeds.^5^ ^,^ ^44^ ^,^ ^45^

Interestingly, *NCED3* expression was also elevated in the *gata5* mutant despite the absence of functional AtGATA5 protein (Figure 2). This counterintuitive result suggests that compensatory regulatory mechanisms are activated in the absence of *AtGATA5*. Loss of *AtGATA5* function may alter chromatin accessibility at the *NCED3* locus, or relieve competition from an *AtGATA5*-dependent repressor, permitting increased *NCED3* transcription through alternative activators. Furthermore, given that *AtGATA5* is regulated by the circadian clock and light signaling,^33-35^ its loss could disrupt circadian gating of *NCED3* expression, resulting in deregulated transcript accumulation.^38^ ^,^ ^44^ The mechanism by which *NCED3* becomes elevated in *gata5* remains to be elucidated, and may involve ABA feedback regulation, altered seed ABA profiles,^43^ ^,^ ^46^ or disrupted primary dormancy release as shown for other ABA pathway mutants.^46^ ^,^ ^47^ Regarding the *AtGATA5* transcript level in *gata5* mutant, the near WT mRNA abundance detected by qRT-PCR is attributable to the nature of the CRISPR/Cas9-induced frameshift mutation, which introduces a premature stop codon that abolishes protein function while leaving transcription at the *AtGATA5* locus unaffected.

In contrast to *NCED3*, *ABI4* was markedly upregulated in *AtGATA5*-OX plants but undetectable in *gata5* (Figure 2), suggesting that *AtGATA5* is required for *ABI4* expression. This is consistent with TAA results showing elevated *ABI4* promoter activity in the presence of the AtGATA5 effector in WT protoplasts (Figure 3), and restored activity upon re-introduction of AtGATA5 into *gata5* protoplasts (Figure 4). ABI4 is an AP2/ERF transcription factor central to ABA-mediated repression of seed germination and early seedling establishment.^16-19^ ABI4 represses GA-responsive genes while reinforcing ABA-responsive elements, sustaining the ABA-dominant state that inhibits germination.^17^ ^,^ ^18^ Furthermore, ABI4 promotes *NCED3* expression through a positive feedback loop,^15^ amplifying ABA biosynthesis. The absence of *ABI4* expression in *gata5* plants, together with restored promoter activity upon AtGATA5 re-introduction, suggests that *AtGATA5* may function as an upstream component of this *ABI4*–*NCED3* regulatory module. This is consistent with the established role of ABI5 as a downstream effector that coordinates ABA responses with post-germination growth arrest.^20^

In *gata5* protoplasts, *NCED3* promoter activity was slightly elevated compared with WT, whereas *ABI4* promoter activity was similar to WT (Figure 4). This finding further supports the conclusion that *AtGATA5* is dispensable for basal *NCED3* transcription but specifically required for *ABI4* activation. The slight elevation in *NCED3* promoter activity in the *gata5* background may reflect compensatory activity of alternative transcriptional activators at the *NCED3* locus, but not at the *ABI4* locus. Identifying the *cis*-regulatory elements within the *NCED3* and *ABI4* promoters directly bound by AtGATA5 through chromatin immunoprecipitation (ChIP) and electrophoretic mobility shift assay (EMSA) would provide mechanistic insight into this differential regulation.

The finding that *AtGATA5* modulates ABA-mediated seed germination extends its functional repertoire beyond the previously described role in SCW biosynthesis.^36^ ^,^ ^48-53^ *AtGATA5* was previously shown to positively regulate NAC-domain transcription factors, master regulators of the SCW biosynthesis pathway,^36^ ^,^ ^51^ ^,^ ^52^ ^,^ ^54^ thereby influencing SCW formation.^36^ ^,^ ^37^ Related GATA factors in this laboratory have demonstrated similar functional versatility: *GATA25* regulates flowering time^30^ and hypocotyl elongation^31^ through CRISPR gain- and loss-of-function analyses, and the poplar ortholog PtrGATA9 activates NAC transcription factors to facilitate interfascicular fiber cell deposition.^32^ At the downstream level, *AtGATA5*-regulated targets such as MYB46 link the GATA-NAC regulatory axis to cellulose synthase gene expression.^48-50^ These findings collectively suggest that the GATA transcription factor family functions as a versatile transcriptional integrator across multiple developmental and hormone-signaling contexts.

**Figure 5.**
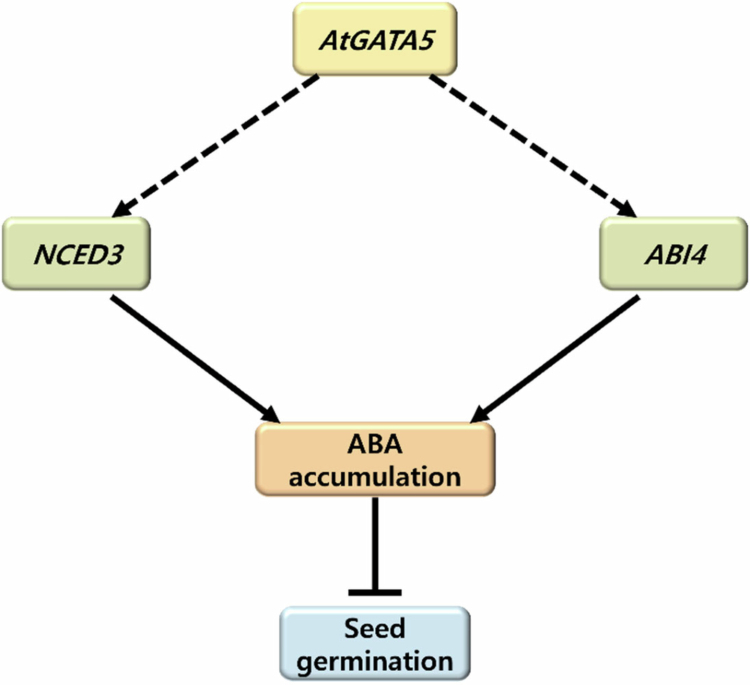
Proposed working model for *AtGATA5*-mediated regulation of ABA-dependent seed germination in *Arabidopsis thaliana*. AtGATA5 is associated with elevated *NCED3* and *ABI4* expression levels as determined by qRT-PCR and TAA. Both *NCED3* and *ABI4* promote ABA accumulation, which in turn inhibits seed germination. Dashed arrows indicate regulatory relationships based on the results.

Taken together, the present data reveal a previously uncharacterized role for *AtGATA5* in ABA-mediated seed germination. *AtGATA5*-OX and *gata5* plants both displayed ABA hypersensitivity, accompanied by elevated *NCED3* and *ABI4* transcript levels and enhanced promoter activities as assessed by TAA. These observations suggest that *AtGATA5*, a GATA-type transcription factor known to be responsive to light and circadian signals,^33-35^ ^,^ ^37^ may function upstream of ABA biosynthesis and signal transduction by promoting the expression of *NCED3* and *ABI4*. Based on these results, we propose a working model in which AtGATA5 facilitates ABA accumulation and signal amplification through a regulatory network involving *NCED3* and the *ABI4*–*NCED3* positive feedback module (Figure 5). However, it should be noted that the current evidence is based on expression analyses and TAA data, and whether AtGATA5 regulates *NCED3* and *ABI4* through direct promoter binding or via intermediate regulators remains to be established.

## Supplementary Material

Supplementary Material Supplementary_material.docx
