# Supplementary material for: AtGATA5 contributes to ABA-mediated seed germination by promoting NCED3 and ABI4 expression in Arabidopsis thaliana
Source: Plant Signal Behav. 2026 Jun 11;21(1):2687956. doi: 10.1080/15592324.2026.2687956 (PMC13271278; doi:10.1080/15592324.2026.2687956)
Supplement: Supplementary Material — Supplementary_material.docx [file KPSB_A_2687956_SM4969.docx]

**Supplementary Table S1.** List of primers used in this study


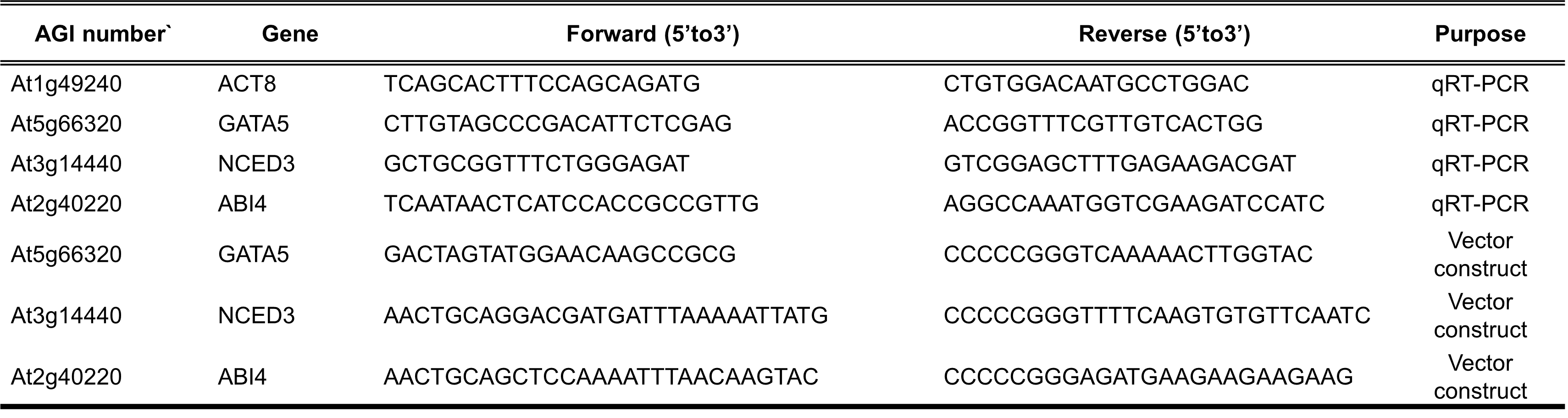


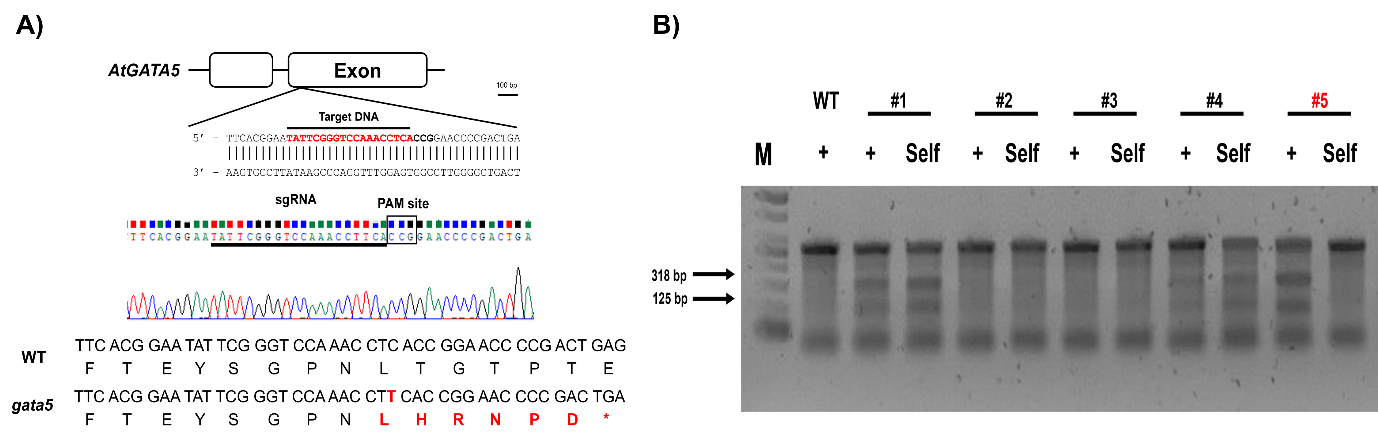


**Fig. S1** Generation and confirmation of the CRISPR/Cas9-mediated *gata5* mutant. (A) Schematic representation of *AtGATA5* showing the sgRNA target site within the exon (top). The gRNA targeted DNA sequence is underlined and the protospacer adjacent motif (PAM) is in the box. The sequencing chromatogram confirms editing at the target locus (middle). Sequence alignment of WT and *gata5* alleles shows a 1 bp insertion (bold, red) resulting in a frameshift and premature stop codon (*) (bottom). Scale bar = 100 bp. (B) T7E1 assay-based genotyping of T2 lines. Cleavage products confirm the presence of the *gata5* mutation. Line #5 was confirmed as homozygous and selected for subsequent analyses.
